# MAP Kinase Phosphatase-2 Plays a Key Role in the Control of Infection with *Toxoplasma gondii* by Modulating iNOS and Arginase-1 Activities in Mice

**DOI:** 10.1371/journal.ppat.1003535

**Published:** 2013-08-15

**Authors:** Stuart Woods, Juliane Schroeder, Helen A. McGachy, Robin Plevin, Craig W. Roberts, James Alexander

**Affiliations:** Strathclyde Institute of Pharmacy and Biomedical Sciences, University of Strathclyde, Glasgow, United Kingdom; National Institute of Health, United States of America

## Abstract

The dual specific phosphatase, MAP kinase phosphatase-2 (MKP-2) has recently been demonstrated to negatively regulate macrophage arginase-1 expression, while at the same time to positively regulate iNOS expression. Consequently, MKP-2 is likely to play a significant role in the host interplay with intracellular pathogens. Here we demonstrate that MKP-2^−/−^ mice on the C57BL/6 background have enhanced susceptibility compared with wild-type counterparts following infection with type-2 strains of *Toxoplasma gondii* as measured by increased parasite multiplication during acute infection, increased mortality from day 12 post-infection onwards and increased parasite burdens in the brain, day 30 post-infection. MKP-2^−/−^ mice did not, however, demonstrate defective type-1 responses compared with MKP-2^+/+^ mice following infection although they did display significantly reduced serum nitrite levels and enhanced tissue arginase-1 expression. Early resistance to *T. gondii* in MKP-2^+/+^, but not MKP-2^−/−^, mice was nitric oxide (NO) dependent as infected MKP-2^+/+^, but not MKP-2^−/−^ mice succumbed within 10 days post-infection with increased parasite burdens following treatment with the iNOS inhibitor L-NAME. Conversely, treatment of infected MKP-2^−/−^ but not MKP-2^+/+^ mice with nor-NOHA increased parasite burdens indicating a protective role for arginase-1 in MKP-2^−/−^ mice. In vitro studies using tachyzoite-infected bone marrow derived macrophages and selective inhibition of arginase-1 and iNOS activities confirmed that both iNOS and arginase-1 contributed to inhibiting parasite replication. However, the effects of arginase-1 were transient and ultimately the role of iNOS was paramount in facilitating long-term inhibition of parasite multiplication within macrophages.

## Author Summary

*Toxoplasma gondii* is a protozoan (single cell) parasite that can be transmitted to humans via infection stages in cat feces or from eating under-cooked meat containing parasite cysts. The disease, which infects some 30% of the world population is a major cause of congenital infection and abortion and can be fatal in immune compromised hosts. Control of parasite growth has been shown to be largely, but not wholly, dependent on the production of nitric oxide (NO) as a result of the enzymatic activity of inducible nitric oxide synthase (iNOS) on the substrate L-arginine. It has been suggested that arginase-1 by depleting the L-arginine store can promote parasite growth. We have recently shown MAP kinase phosphatase-2 (MKP-2) to increase iNOS and decrease arginase-1 levels and mice lacking this gene were found to be more susceptible to *T. gondii*. Inhibition of iNOS activity in genetically intact mice demonstrated it was of paramount importance in controlling parasite growth. Surprisingly, however, inhibition of arginase-1 in MKP-2^−/−^ mice demonstrated arginase-1 was also protective. Significantly *T. gondii* cannot make its own arginine and requires this from the host cell and consequently iNOS and arginase-1 can work together to starve the parasite of this essential metabolite.

## Introduction

*Toxoplasma gondii* is an obligate intracellular protozoan parasite of significant public health importance, being a major cause of congenital infection and abortion as well as a significant and often fatal infection in immune compromised hosts. The early acute stage of infection is characterized by widespread tachyzoite dissemination and tissue damage. The rapid onset of immunity, initiated in large part by the well characterized *T. gondii* pathogen associated molecular patterns (PAMPS) controls parasite replication [1]–[4], and results in the life-long chronic stage of infection associated with encystment of the parasites in skeletal muscle and the central nervous system [reviewed in 5]. Protection against acute disease is mediated primarily by the interaction of neutrophils, dendritic cells, macrophages and natural killer (NK) cells that as part of the innate response not only limits parasite growth, but initiates an effective cytotoxic CD8^+^ T cell response that is responsible for long-term protection and prevention of encephalitis via IFN-γ production [6]–[9]. The mechanisms by which IFN-γ, the major effector cytokine mediating resistance during *T. gondii* infection, promotes anti-*toxoplasma* activity are not yet fully clear and vary between host species studied. Several IFN-γ-regulated genes including iNOS [6], [10], indoleamine 2,3 dioxygenase (IDO) [11], [12], and more recently, p47 GTPases, have been implicated in playing significant roles in mediating these protective responses [13]–[21].

Despite being essential to control parasite replication, an overactive type-1 response and overproduction of IFN-γ, TNF-α and NO can result in severe pathology and death. Consequently, protective immunity to *T. gondii* that needs to effectively control parasite proliferation without excessive inflammation is dependent on the regulation of type-1 responses by Th2 cells and Treg cells [22]–[29]. An overactive type-2 response could equally promote enhanced parasite proliferation and host death and indeed alternative macrophage activation has been shown to promote parasite growth [30]. Interestingly, the classic hallmark indicator of alternative macrophage activation, arginase-1, can be induced innately by *T. gondii* apparently via both STAT-6 dependent [30]–[32] and independent mechanisms [30], [33]. Paradoxically, arginase-1 has been associated with enhancing infection with *T. gondii* by competing with iNOS for their common substrate L-arginine [33] and promoting parasite replication by providing the polyamines needed for cell division [32]. Conversely arginase-1 might also limit parasite growth by starving the parasite of the L-arginine it requires for this process [31], [34].

A recent study has demonstrated that the dual specific phosphatase, MKP-2 is not only a negative regulator of macrophage arginase-1 but also a positive regulator of iNOS expression [35]. Furthermore MKP-2^−/−^ C57BL/6 mice have been found to display enhanced susceptibility to the intracellular parasite *Leishmania mexicana*. Susceptibility in MKP-2^−/−^ mice was in large part due to enhanced parasite growth that could be reversed by inhibiting arginase-1 activity. Given the importance of NO production as well as the apparently contradictory roles of arginase-1 in murine *T. gondii* infections it is likely that the role of MKP-2 in *T. gondii* infection would be one of major significance. We consequently studied the course of *T. gondii* infection in MKP-2^−/−^ and MKP-2^+/+^ C57BL/6 mice. We identified that MKP-2 deficiency results in increased susceptibility to *T. gondii* infection and that this correlated strongly with impaired iNOS activity *in vivo*. Conversely we demonstrated an arginase-1 dependent mechanism responsible for control of parasite growth that functioned to partially protect the host independently of iNOS mediated effects.

## Materials and Methods

### Ethics statement

All animal procedures conformed to guidelines from The Home Office of the UK Government. All work was covered by two Home Office licences: PPL60/3929, “mechanism of control of parasite infection” and PPL60/3439,“genetic models of cancer and inflammation” with approval by the University of Strathclyde ethical review panel.

### Mice

MKP-2^+/+^ and MKP-2^−/−^ mice on a C57BL/6 background were bred and maintained and all experiments carried out in house at the Strathclyde Institute of Pharmacy and Biomedical Sciences, Glasgow, UK. Six to eight week old, male mice were used for infection and aged matched within each experiment.

### Maintenance and infection of *Toxoplasma gondii* Beverly strain

Beverley cysts were maintained *in vivo* by intraperitoneal (i.p.) passage of infective brain tissue homogenates through outbred CD1 albino mice. For experimental infections MKP-2^+/+^ and MKP-2^−/−^ mice were infected i.p. with 10 tissue cysts in 200 µl sterile PBS.

### Maintenance of transfected *Toxoplasma gondii* Prugniaud strains

Tachyzoites were routinely maintained in confluent human foreskin fibroblasts (HFFs) grown in DMEM complete medium comprising; Dulbecco's Modified Eagle Medium (DMEM) containing L-glutamine (Invitrogen, UK), 10% foetal calf serum (PAA, UK), 100 U/ml penicillin (Cambrex Bioscience, Veniers, Belgium), 100 µg/ml streptomycin (Cambrex Bioscience, Veniers, Belgium) and 50 U/ml amphotericin B (Cambrex Bioscience, Veniers, Belgium) at 37°C in 5% CO_2_.

### Bioluminescent imaging

*In vivo* parasite burden was assessed using bioluminescent imaging using type II Prugniaud *T. gondii* transfected with the firefly luciferase [36]. The light data was quantified using Living Image software (Caliper Life Science)

Briefly, intracellular FLUC *T. gondii* was harvested from *in vitro* culture. Culture media was removed and the HFF monolayer washed once with sterile PBS (Lonza, UK) prior to disruption with a cell scraper and harvesting in 10 ml sterile PBS. The intracellular parasites were then released from the host cells by passage through a 21 gauge needle and centrifuged at 1200 rpm for 10 minutes. The bioluminescent activity of the FLUC *T. gondii* was quality controlled for each experiment prior to infection using a standard curve in a black-walled 24 well plate by *in vitro* imaging using the IVIS Spectrum (Caliper Life Sciences) (Figure S1). Mice were then infected with 20,000 tachyzoites i.p. in a volume of 400 µl sterile PBS. FLUC *T. gondii* infected mice were imaged using the IVIS Spectrum (Caliper Life Sciences) to determine parasite burden. Mice to be imaged were given 150 mg/kg of D-luciferin potassium salt solution i.p. prior to anaesthesia with isoflurane. For optimal imaging, 1 minute exposures were taken with medium binning, 20 minutes post luciferin injection. For *ex vivo* imaging of the brain, mice were injected with 150 mg/kg of D-luciferin potassium salt solution. After 10 minutes the mice were sacrificed by CO_2_ inhalation. The brains were removed and soaked in 15 mg/ml D-luciferin potassium salt, dissolved in warmed RMPI 1640, for a further 10 minutes before being imaged.

### Purification of *Toxoplasma* lysate antigen

RH tachyzoites where harvested from acutely infected BALB/c mice, by intra-peritoneal washout and lysate antigen (TLA) was prepared as described previously [37].

### Cytokine ELISA

Spleen cell suspensions from infected mice were prepared as described previously [24]. For re-stimulation, 2×10^5^ cells were incubated with 5 µg TLA in a total volume of 200 µl for 72 h at 37°C in 5% CO_2_. Supernatants were then used for IFN-γ, IL-5, IL-6 and IL-10 ELISA, using paired antibodies from BD Bioscience. Absorbance was the read at 405 nm using a microtitre plate reader (Spectramax 190, Molecular Devices, USA). Concentrations were determined against standard curves for each cytokine (R&D Systems).

### Serum nitric oxide

Serum nitric oxide levels were determined by Griess assay. Blood was collected by cardiac puncture and cells removed by centrifugation at 13,000 rpm for 10 minutes. Protein was removed from the serum by adding ZnSO_4_ to a final concentration of 15 mg/ml, vortexing thoroughly and centrifuged at 13,000 rpm for 10 minutes. The supernatant was retained for the Griess assay. Griess reagent (equal volumes of 2% sulphanilamide in 5% H_3_PO_4_ and 0.2% Napthylene diamine HCL in ddH_2_O) was added to samples and standards in a 96 well plate and incubated in the dark for 10 minutes. Absorbance was read at 540 nm and serum nitrite concentrations were calculated against a standard curve.

### *In vivo* iNOS and Arginase-1 inhibition

To inhibit iNOS and Arginase-I *in vivo*, mice received intraperitoneal doses of N (G)-nitro-L-arginine methyl ester (200 mg/kg, L-NAME, Sigma UK) or N^ω^-hydroxy-nor-Arginine (100 µg nor-NOHA, Merck, UK), respectively [38], [39]. Treatment commenced one day prior to infection.

### Generation of murine macrophages

Bone marrow derived macrophages were cultured by flushing the femurs and tibiae of 6 week old MKP-2^+/+^ or MKP-2^−/−^ mice with DMEM. Cells were then cultured in DMEM containing 20% heat inactivated FCS, 30% L-cell conditioned medium, 5 mM L-glutamine, 100 U/ml penicillin and 100 µg/ml streptomycin and incubated at 37**°**C for 10 days [35]. After this time adherent cells were harvested and then seeded into either 12 well plates (1×10^6^ cells/ml) or black-walled 96 well plates (0.5×10^5^).

### Macrophage infection with *T. gondii*

For infection, 5×10^4^ bone marrow derived MKP-2^+/+^ or MKP-2^−/−^ macrophages were seeded into black-walled 96 well plates in complete phenol red free RPMI 1640 medium. The cells were infected with 5×10^4^ type II Prugniaud strain *T. gondii* tachyzoites, expressing YFP (Donated by Marcus Meissner, University of Glasgow). Wells were made up to a final volume of 200 ml. Macrophages were treated with LPS (100 ng/ml), IFN-γ (100 U/ml), 50 µM nor-NOHA or 5 mM L-NAME as appropriate. Levels of YFP expression were assayed at 24, 48 and 72 h using the transillumination feature of the IVIS Spectrum (Caliper Lifescience). An excitation wavelength of 500 nm and emission wave of 540 nm was used. The light data was quantified using Living Image software (Caliper Lifescience) using the uninfected macrophages as background controls.

### Flow cytometry

For re-stimulation, 1×10^6^ splenocytes were incubated with 5 mg/ml TLA, ionomycin and phorbol-13-myristate-12-acetate (PMA, Sigma, UK) (0.5 µg/ml and 10 ng/ml, respectively) for 6 h at 37°C and 5% CO_2_. After 3 h incubation Brefeldin A (Sigma) was added for a further 3 h at a final concentration of 10 ng/ml. Cells were then harvested for staining and incubated with Fc-Block (5 µg/ml αCD16/CD32, BD Bioscience, 1% mouse serum in RPMI) for 10 minutes at room temperature, cells were stained for 1 hour at 4°C with αCD3-PerCP, αCD4-APC-H7 (BD Biosciences) and αCD8-Alexa488 (ebioscience). Cells were fixed using Fix & Perm (Life Technologies) following manufacturer's instructions. Intracellular cytokine staining was performed using αTNF-PE and αIFN-γ-APC (eBioscience). A total of 500,000 events per sample were acquired on a BD FACSCanto and data analysis was carried out using FlowJo software.

Natural killer or NKT cells from intraperitoneal exudates (1×10^6^) from infected mice were surface stained with αPanNK-APC and αCD3-PerCP-eFluor710 before fixing and permeabilisation. Intracellular staining for IFN-γ was performed using αIFN-γ-PE or the respective isotype control, rat IgG1,κ-PE (all eBioscience). A total of 200,000 events was recorded and analysed using Kaluza software (Beckman Coulter). All values were normalized to their respective isotype control.

### Measurement of Arginase-1 expression and activity

Cell lysates (20 µg protein/lane) were separated by 10% SDS-PAGE and transferred onto a nitrocellulose membrane and probed for Arginase-1 as previously described [35]. Arginase activity from murine BMD-macrophages was measured using an assay based on a reaction with α-isonitrosopropiophenon (ISPF) as described previously [35].

### Preparation of cDNA

Peritoneal exudates from mice were counted and 1×10^6^ cells from each sample taken for RNA extraction using RNeasy Mini Kit (Qiagen, UK) following manufacturer's instructions. For each reaction, 2 µg of total RNA and 300 ng of random primers (Promega) were incubated at 65°C for 5 minutes. Following a 10 minute incubation at room temperature 2 µl of AffinityScript reverse transcriptase (RT) buffer (Agilent Technologies), 10 mM dithiothreitol, 4 mM dNTP mix (Promega) and 1 µl of AffinityScript multiple RT (Agilent Technologies) were added to each sample. These were then incubated for 10 minutes at 25°C, 1 hour at 50°C and 15 minutes at 70°C.

### Quantitative real-time PCR (qRT-PCR)

qRT-PCR experiments were conducted on the Stratagene Mx3000p system (Stratagene, Agilent). Each reaction consisted of 5 µl Brilliant III Ultra-fast SYBR Green QPCR Master Mix (Agilent Technologies), 3.5 µl of molecular grade water (Life Technologies), 25 pmol of the forward and reverse primers (Table S1), and 1 µl of the cDNA template. PCR reactions were carried out with the following thermoprofile: one cycle at 95°C for 10 minutes, 40 cycles of 20 seconds at 95°C, 20 seconds at 64°C and 30 seconds at 70°C. Relative gene expression was calculated based on the ΔCT for each gene, normalized to that of the housekeeping gene (*TBP*).

### Statistics

Statistical analysis was performed using GraphPad Prism Program (Version 5.0, GraphPad Software, California). All results are shown as standard error of the mean (SEM). Statistically significant differences were determined using students t-test for parametric and Mann Whitney U test for non-parametric data, respectively. P values equal or below 0.05 were considered significant.

## Results

### MKP-2 deficiency results in increased susceptibility following infection with *T. gondii*

**Figure 1:**
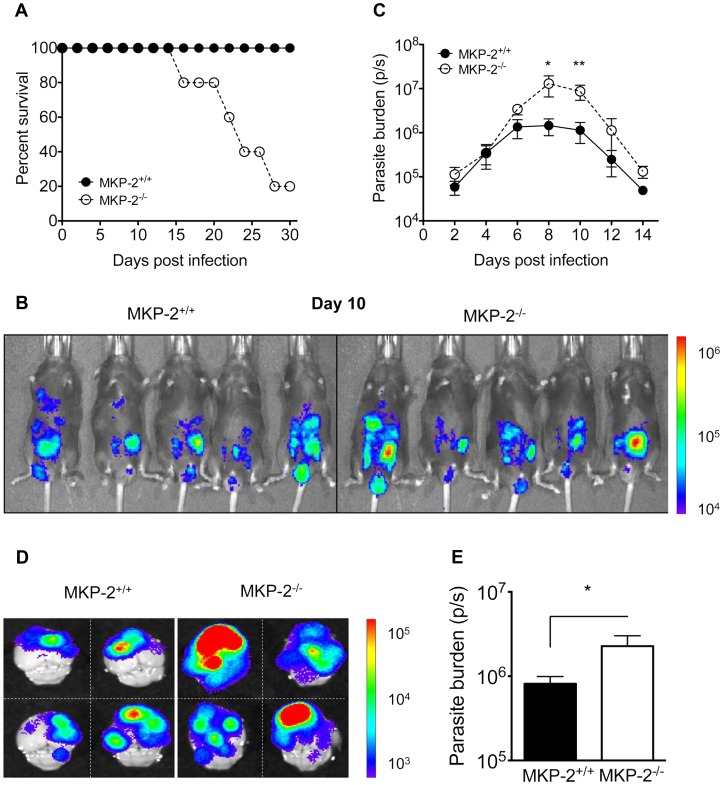
MKP-2 deficiency results in increased susceptibility to *T. gondii* infection. Mice were infected with 10 Beverley tissue cysts intraperitoneally and mortality monitored (A). For *in vivo* bioluminescent imaging and parasite quantification, mice were infected with 20,000 type II Prugniaud tachyzoites, expressing firefly luciferase. Mice were imaged as outlined in the methods (B). Parasite burdens were determined by measuring whole body Total Flux (Photons/second) using Living Image 4.0 (C). Each value represents the mean of 5 animals per experimental group ± SEM. On day 30 post-infection brains were imaged *ex vivo* as outlined in the methods (D). Chronic parasite burden was determined by measuring Total Flux (photons/second) from each brain (E). Each value represents the mean of four animals per experimental group ± SEM. All experiments were carried out on at least two occasions.

Intraperitoneal infection with 20 cysts of the Beverley (type-II) strain of *T. gondii* resulted in significant mortality, typically between 80–100%, 15–25 days post-infection in MKP-2^−/−^, but not MKP-2^+/+^ mice (Figure 1A). In order to determine whether this was associated with any inability of the MKP-2^−/−^ mice to control parasite growth, MKP-2^−/−^ and MKP-2^+/+^ mice were infected i.p. with 20,000 type-II strain Prugniaud-FLUC tachyzoites and parasite burdens measured at 2 day intervals by bioluminescent imaging (Figure 1B and C). At days 8 (Figure 1B) and 10 the bioluminescent intensity and consequently parasite burdens were significantly greater (p<0.031 and p<0.0079 respectively) in MKP-2^−/−^ compared with MKP-2^+/+^ mice (Figure 1C). Greater parasite numbers in infected MKP-2^−/−^ as opposed to MKP-2^+/+^ mice during acute infections were confirmed by qRT-PCR (Figure S2) The increased susceptibility of MKP-2^−/−^ mice infected with Prugniaud-FLUC tachyzoites compared with their wild-type counterparts was also evident during the chronic phase of the disease: mortality was typically 40–60% in MKP-2^−/−^ mice and by day 30 post-infection the excised brains of MKP-2^−/−^ mice were revealed to have significantly higher parasite burdens (p<0.02) than similarly infected MKP-2^+/+^ animals (Figure 1D and E). Increased parasite burdens in infected MKP-2^−/−^ compared with MKP-2^+/+^ mice were also evident in other tissues at this late stage (Liver: MKP-2^−/−^ 4.76×10^5^±4.7×10^3^p/s, MKP-2^+/+^ 3.85×10^5^±4.6×10^4^p/s; Kidney: MKP-2^−/−^ 1.31×10^6^±4×10^4^p/s, MKP-2^+/+^ 4.76×10^5^±1.3×10^5^p/s p<0.05).

### MKP-2^−/−^ mice infected with *T. gondii* do not have an impaired immune response

**Figure 2:**
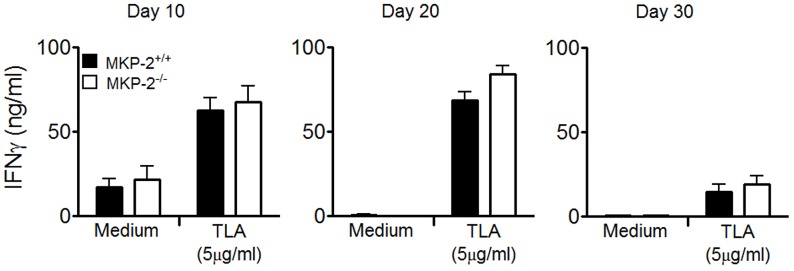
MKP-2^−/−^ and MKP-2^+/+^ splenocyte IFN-γ production was similar through the acute and chronic stages of infection. Splenocytes from *T. gondii* infected mice were stimulated with TLA (5 µg/ml) and the supernatants assessed for IFN-γ by ELISA. Each value represents the mean of four animals per experimental group ± SEM. All experiments were carried out on at least two occasions.

**Figure 3:**
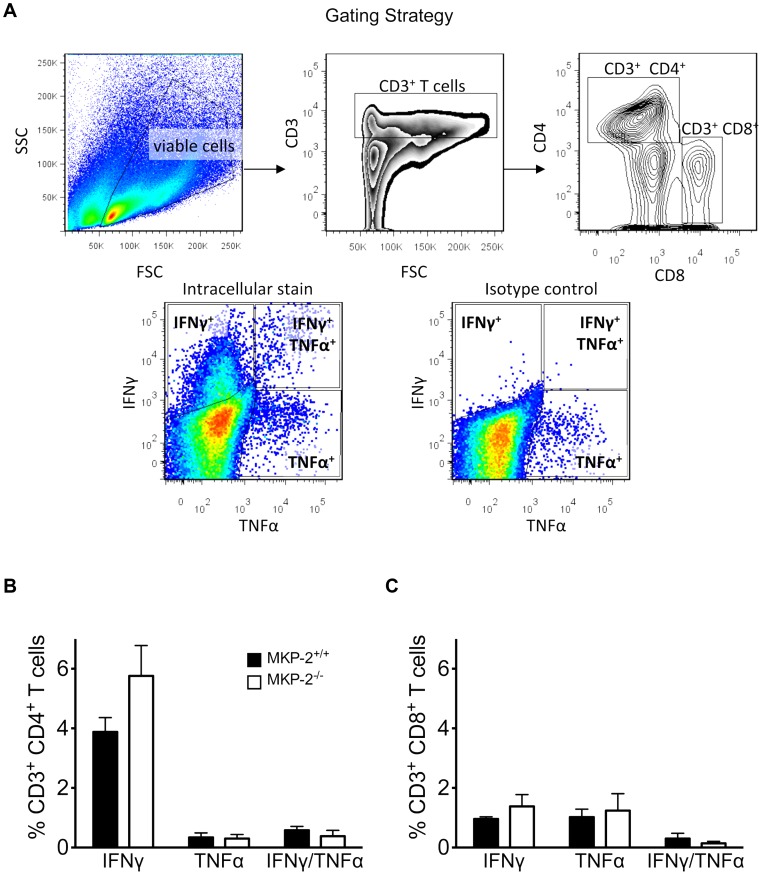
MKP-2 deletion does not impair T cell responses during infection with *T. gondii*. T cell responses were determined by flow cytometry. Cells were surface-stained for CD3, CD4 and CD8 and intracellular for IFN-γ and TNF-α. Live cells were gated on forward (FSC) versus side scatter (SSC). T cells were first gated for CD3 and then sub-gated for either CD4 or CD8 and subsequently their respective antigen-specific intracellular cytokine production, following stimulation with TLA (10 µg/ml). The specificity of the intracellular staining was ensured by analysing the respective isotype controls and normalizing samples accordingly (A). Populations of CD3^+^ CD4^+^ T cells (B) and CD3^+^ CD8^+^ T cells (C) single or double positive for IFN-γ and TNF-α were determined using FlowJo software. Each value represents the mean of four animals per experimental group ± SEM. All experiments were carried out on at least two occasions.

In order to determine whether the increased susceptibility of MKP-2^−/−^ mice infected with *T. gondii* was the result of an impaired adaptive immune response spleens were removed and splenocytes stimulated with TLA at days 10, 20 and 30 post-infection and IFN-γ, IL-4, IL-5 and IL-10 production measured in the supernatants. There were generally no differences between the ability of splenocytes from MKP-2^−/−^ or MKP-2^+/+^ mice infected with *T. gondii* to produce cytokines when stimulated with parasite lysate antigen. This was consistent through both acute and chronic infection for the primary mediator of parasite control IFN-γ (Figure 2). There was also no difference in the absolute number of cytokine producing cells between MKP-2^−/−^ and MKP-2^+/+^ mice (Figure S3). Flow cytometry analysis (Figure 3) of splenocytes from MKP-2^−/−^ and MKP-2^+/+^ mice day 10 post-infection either under resting conditions, following antigen stimulation, or following treatment with ionomycin and PMA revealed no differences in the overall numbers of CD4^+^ or CD8^+^ T cells. In addition no differences in the frequencies of CD4^+^ (Figure 3B) or CD8^+^ (Figure 3C) T cells producing IFN-γ, or TNF-α, or both IFN-γ and TNF-α (Figure 3) were noted. In addition, while no IFN-γ was detected in NK cells equivalent amounts of NKT cells producing IFN-γ were detected from both infected MKP-2^−/−^ and MKP-2^+/+^ mice (Figure S4). Consequently, there was little evidence that MKP-2^−/−^ mice were limited in their ability to mount a type-1 response. Furthermore we examined the expressions of IFN-γ dependent GTPases by qRT-PCR and found these to be comparable in both infected MKP-2^−/−^ and MKP-2^+/+^ mice (Figure S2).

### MKP-2^−/−^ mice infected with *T. gondii* have decreased systemic nitrite levels and increased tissue arginase-1 expression

**Figure 4:**
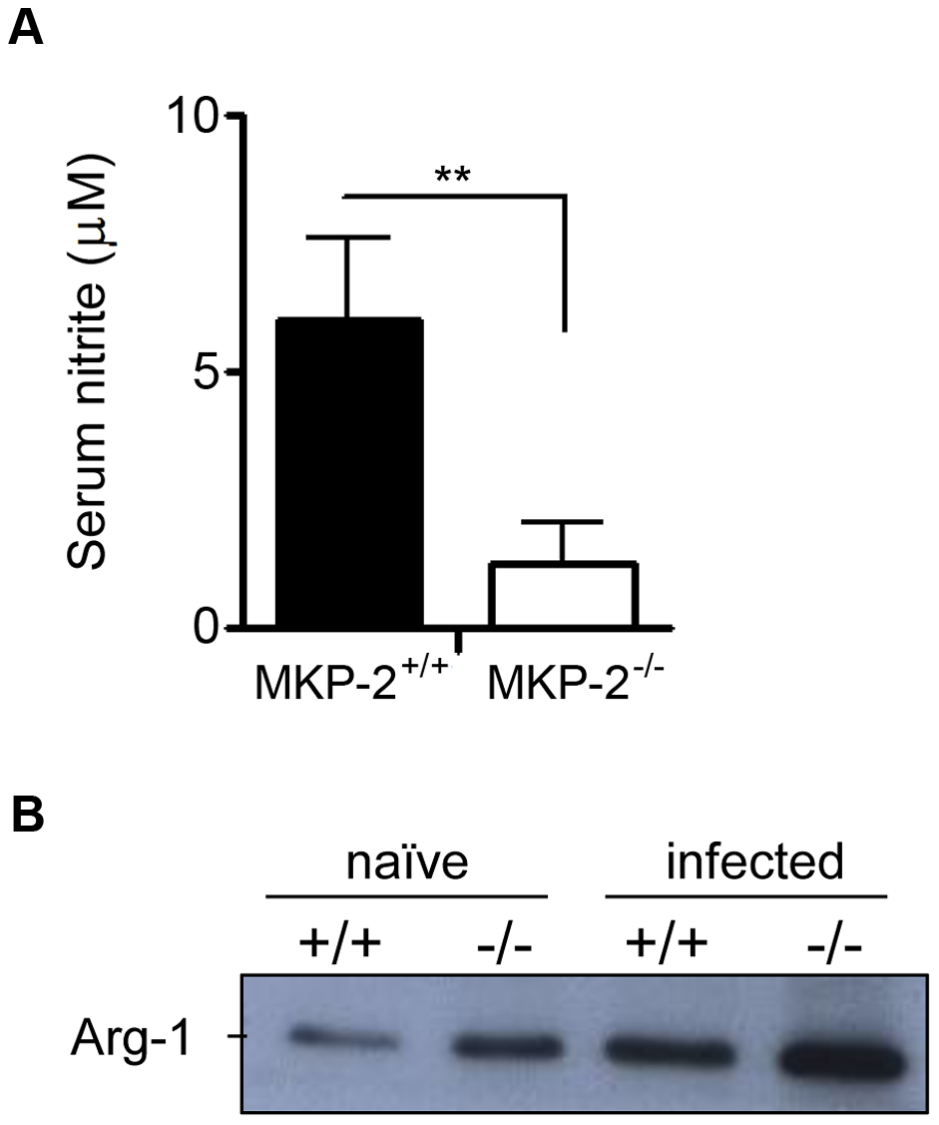
Systemic serum nitrite levels are reduced and tissue arginase-1 expression is enhanced in MKP-2^−/−^ compared with MKP-2^+/+^ mice infected with *T. gondii*. Serum from infected animals was assessed for its nitrite content by Griess assay. Each value represents the mean from ten animals per experimental group ± SEM (A). Splenocyte cell lysates were prepared and assayed for Arginase-1 by western blot. Cells were lysed in sample buffer and protein concentrations measured for each mouse. Equal amounts/animal were pooled and 20 µg loaded for each lane (B). **P<0.005. All experiments were carried out on at least two occasions.

Serum nitrite levels were significantly higher (p<0.0028) in MKP-2^+/+^ mice compared with MKP-2^−/−^ at day 10 post-infection coincident with the noted inverse differences in *T. gondii* parasite burdens between MKP-2^−/−^ and MKP-2^+/+^ mice (Figure 4A). Nitrite levels were below the level of detection in both non-infected MKP-2^−/−^ and MKP-2^+/+^ mice. At the same time spleen arginase-I expression was higher in MKP-2^−/−^ compared with MKP-2^+/+^ mice in both, the uninfected as well as in the *T. gondii* infected groups (Figure 4B). Infection with *T. gondii* in general increased splenic arginase-I levels in all mice independent of the genotype (Figure 4B).

### Inhibition of NO production by L-NAME enhances the susceptibility of MKP-2^+/+^ but not MKP-2^−/−^ mice to *T. gondii* infection

**Figure 5:**
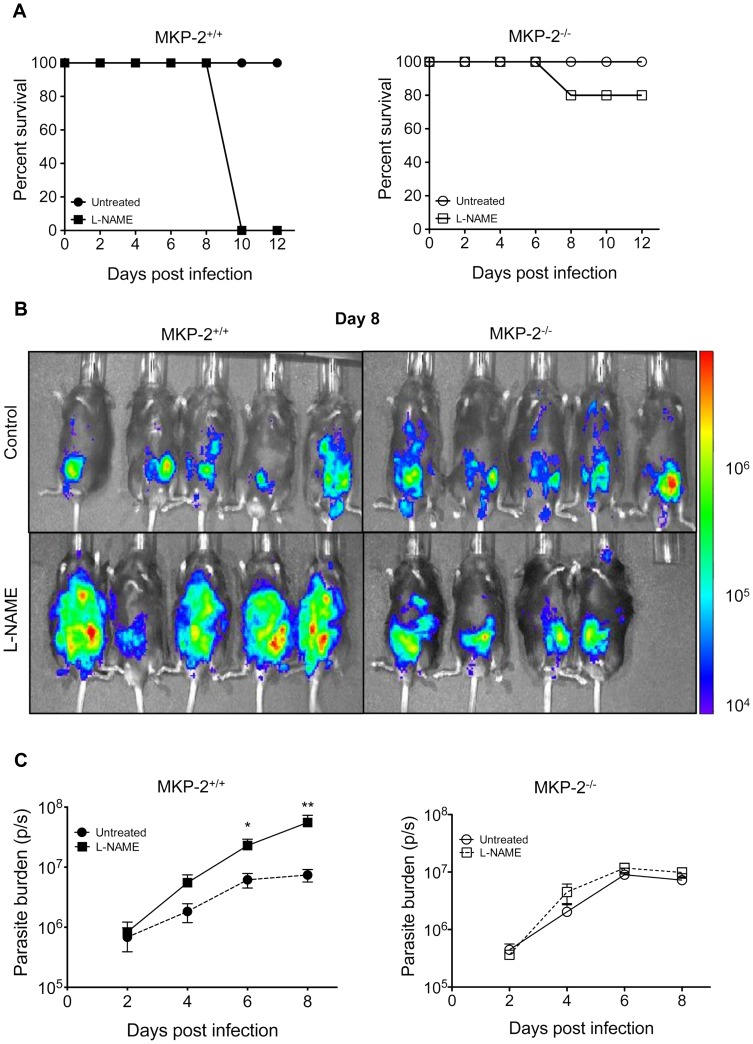
NO inhibition by L-NAME enhances susceptibility of MKP-2^+/+^ but not MKP-2^−/−^ mice to *T. gondii* infection. Mice were pre-treated with L-NAME and subsequently treated with L-NAME (100 mg per mouse) daily following infection with 20,000 Prugniaud tachyzoites expressing firefly luciferase. Mortality was measured over 12 days (A). Mice were imaged every second day. (B) Represents day 8 post-infection. The parasite burden was determined by measuring the total flux (photons/second) for each group (C). Each value represents the mean of five mice per group ± SEM. *P<0.05. All experiments were carried out on at least two occasions.

In order to determine whether the enhanced NO production by *T. gondii* infected MKP-2^+/+^ mice over their MKP-2 deficient counterparts was responsible for their increased resistance to parasite growth and survival *in vivo*, NO production was inhibited in infected mice by intraperitoneal injection with the iNOS inhibitor L-NAME. While L-NAME treatment of infected MKP-2^+/+^ mice resulted in 100% mortality by day 10 post-infection, surprisingly the majority of L-NAME treated infected MKP-2^−/−^ mice survived until day 12 post-infection (Figure 5A). No nitrite was detectable in L-NAME treated mice. All non-treated infected MKP-2^−/−^ and MKP-2^+/+^ mice survived until day 12 (Figure 5A) and in agreement with previous studies at day 10 there were significantly more parasites in MKP-2^−/−^ mice (p<0.03). Measurement of bioluminescence (Figure 5B) allowed quantification of parasite burdens and demonstrated a significant increase in parasite growth at days 6 and 8 post-infection (day 6 p = 0.017, day 8 p = 0.005) in L-NAME treated as opposed to non-treated MKP-2^+/+^ mice (Figure 5C). By comparison treatment of infected MKP-2^−/−^ mice with L-NAME did not significantly alter parasite growth (Figure 5C).

### Inhibition of arginase-1 activity by nor-NOHA enhances the susceptibility of MKP-2^−/−^ but not MKP-2^+/+^ mice to *T. gondii* infection

**Figure 6:**
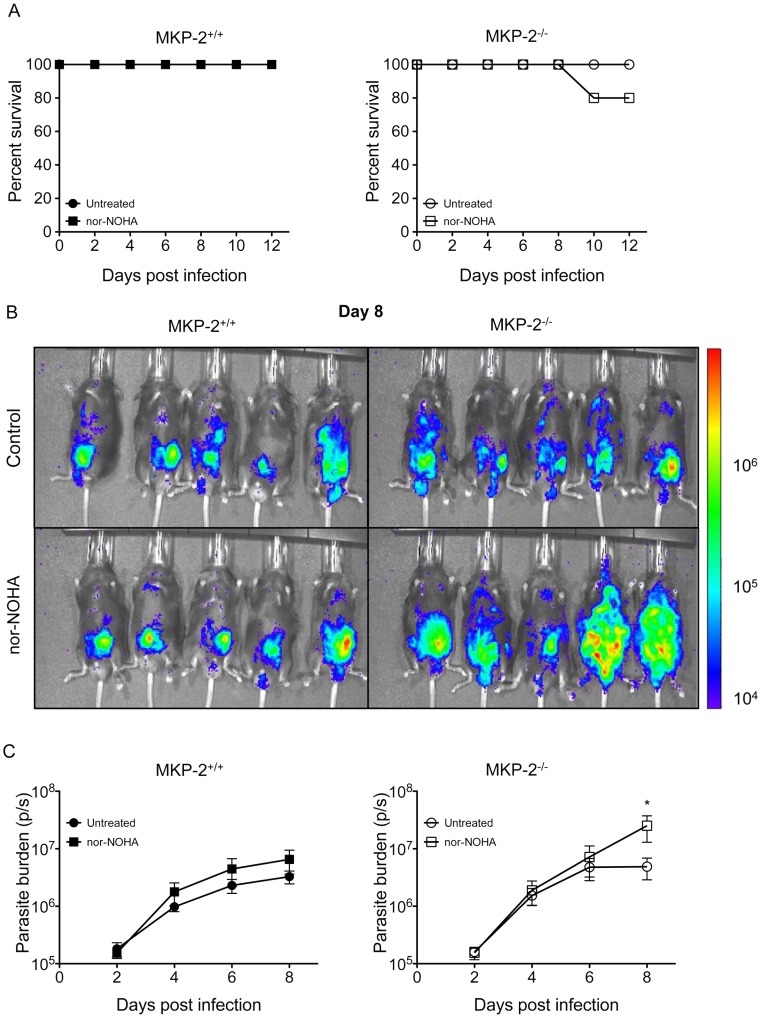
Arginase-1 inhibition by nor-NOHA enhances susceptibility of MKP-2^−/−^ but not MKP-2^+/+^ mice to *T. gondii* infection. Mice were pre-treated with nor-NOHA (200 mg/kg) and treated daily following infection with 20,000 FLUC Prugniaud tachyzoites (A). Mice were imaged every second day post-infection. (B) Represents day 8 post-infection. Total flux (photons/second) was determined for each animal to determine parasite burden (C). Each value represents the mean of five mice per group ± SEM. *P<0.05. All experiments were carried out on at least two occasions.

Unlike their wild-type counterparts, inhibition of NO production did not increase parasite growth or the early mortality of MKP-2^−/−^ mice infected with *T.gondii*. This implied an alternative mechanism of controlling early infection in the absence of MKP-2. As *T. gondii* is an L-arginine auxotroph [40] we suspected that over expression of arginase-1 in MKP-2^−/−^ mice could also be playing a protective role. To determine the possibility that arginase-1 expression might arrest parasite growth through arginine depletion, infected MKP-2^−/−^ and MKP-2^+/+^ mice were treated with the arginase-1 inhibitor nor-NOHA. Nor-NOHA did not significantly increase mortality in either MKP-2^−/−^ nor MKP-2^+/+^ mice infected with *T. gondii* compared with non-drug treated controls (Figure 6A). However, measurement of bioluminescence (Figure 6B) demonstrated significantly greater (p = 0.02 parasite burden day 8 post-infection in nor-NOHA treated as opposed to non-treated MKP-2^−/−^ mice (Figure 6C). This is despite nor-NOHA treatment up-regulating iNOS activity in MKP-2^−/−^ mice as measured by increased nitrite production by as much as ×2. By comparison nor-NOHA treatment had little effect on parasite burden during early *T. gondii* infection in MKP-2^+/+^ mice (Figure 6C).

### MKP-2 deficiency does not render macrophages more susceptible to infection with *T. gondii*

**Figure 7:**
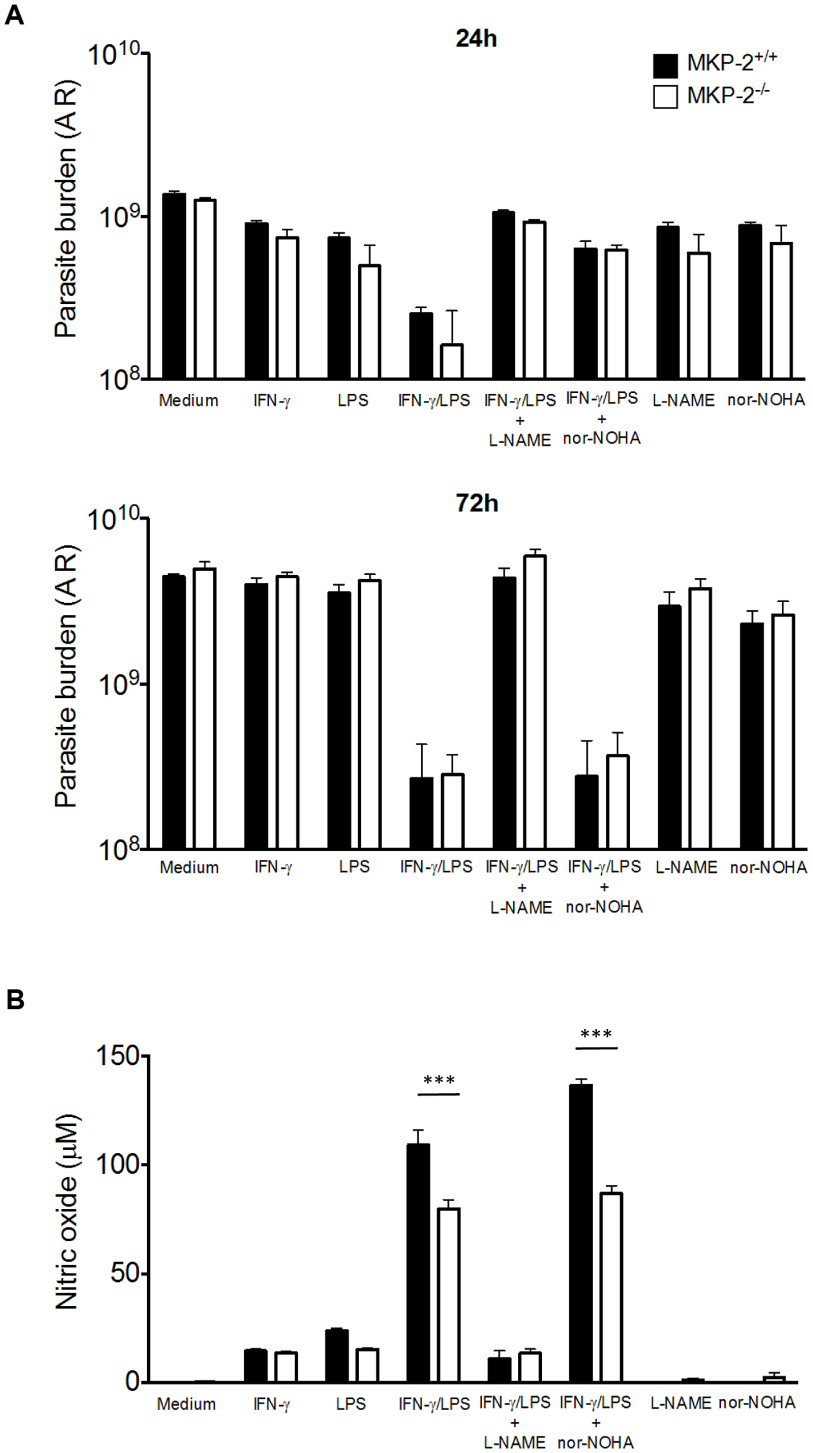
MKP-2 deficient macrophages display deficient iNOS activity but do not display an increased susceptibility to infection. BMD macrophages were treated as appropriate with L-NAME, nor-NOHA, LPS and IFN-γ and subsequently infected with Prugniaud tachyzoites expressing YFP. After 24 and 72 h parasite burdens was determined by measuring YFP fluorescence (A). Supernatants from cultures were assayed for nitrite content by Griess assay (B). Each value represents four replicates ± SEM. ***P<0.005. All experiments were carried out on at least two occasions.

**Figure 8:**
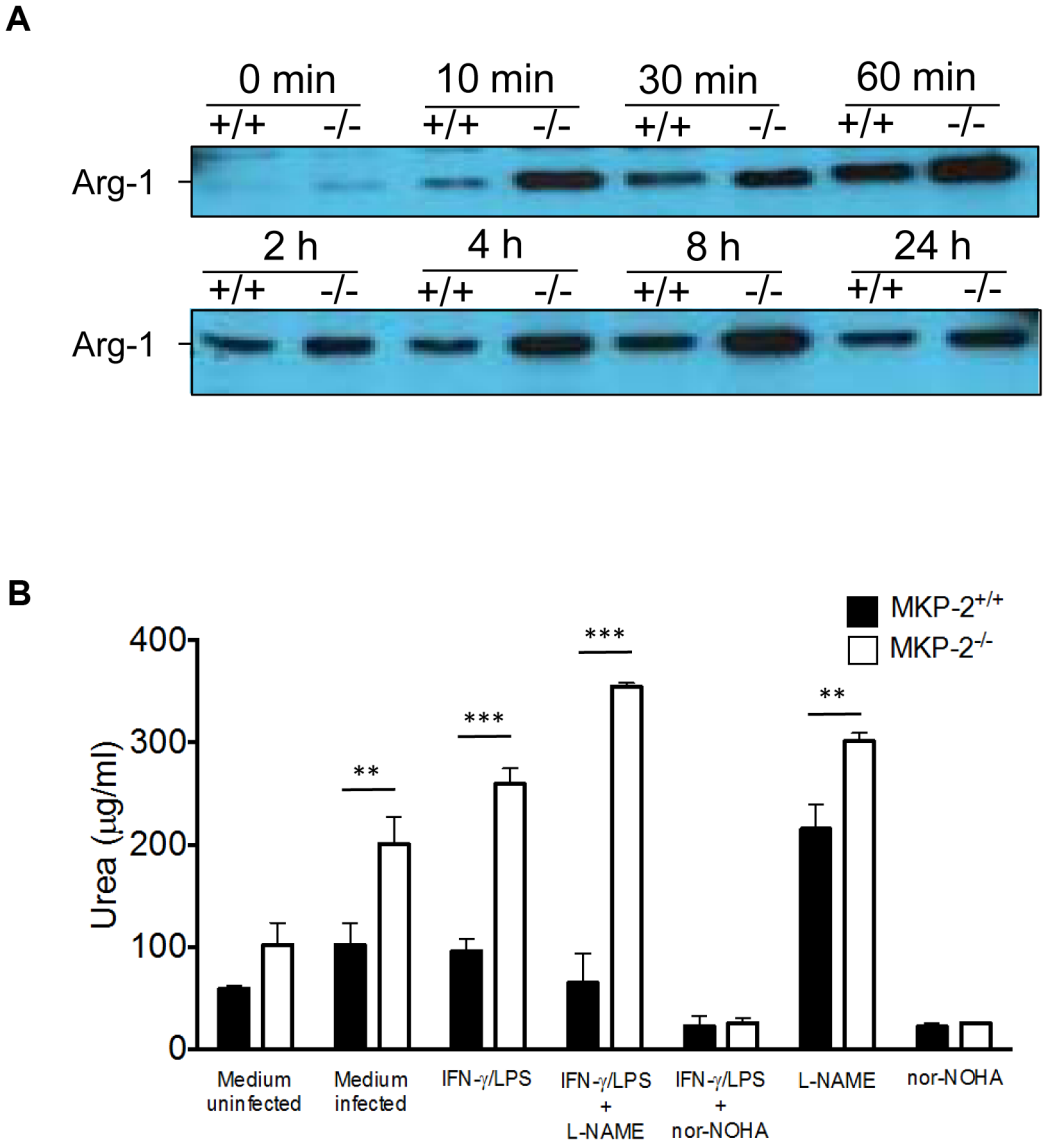
MKP-2 deficient macrophages display enhanced arginase-1 expression and activity. BMD macrophage cell lysates were examined for arginase-1 expression following infection (A). BMD macrophages were treated as appropriate with L-NAME, nor-NOHA, LPS and IFN-γ and subsequently infected with Prugniaud tachyzoites expressing YFP (B). At 24 hours post-infection supernatants were assayed for arginase-1 activity as described in Methods. Each value represents four replicates ± SEM. **P<0.01 ***P<0.005.

We next determined whether the increased susceptibility of MKP-2^−/−^ mice to infection with *T. gondii* was a result of their macrophages being more permissive to parasite growth. MKP-2^−/−^ and MKP-2^+/+^ bone marrow derived macrophages were infected with YFP expressing Prugniaud *T. gondii* tachyzoites at a 1∶1 ratio. In addition the macrophages were either treated with LPS and IFN-γ with or without L-NAME or nor-NOHA. Parasite growth was then determined by assessing YFP fluorescence. At 24 hours, through to 72 hours post-infection parasite growth was similar in non-stimulated MKP-2^−/−^ and MKP-2^+/+^ macrophages (Figure 7A). In addition, following LPS/IFN–γ stimulation parasite growth was significantly, and equally, reduced over the non-treated macrophages (p = 0.0001) and this was irrespective of whether the macrophages were derived from MKP-2^+/+^ or MKP-2^−/−^ mice (Figure 7A). Treatment of LPS/IFN–γ activated MKP-2^−/−^ and MKP-2^+/+^ macrophages with L-NAME partially ablated their ability to control parasite replication at 24 hours and totally ablated their ability to control parasite growth through 48 hours and 72 hours post-infection (p<0.0001) (Figure 7A). Conversely, treatment of LPS/IFN-γ activated MKP-2^−/−^ and MKP-2^+/+^ macrophages with nor-NOHA partially ablated their ability to control parasite growth at 24 hours (MKP-2^+/+^ p = 0.005 and MKP-2^−/−^ p = 0.006 respectively) but not at 48 or 72 hours post-infection (Figure 7A). While LPS/IFN-γ activation stimulated NO production, as measured by nitrite production 72 hours post-infection in the supernatants of *T. gondii* infected MKP-2^−/−^ and MKP-2^+/+^ macrophages, this was significantly higher in the infected MKP-2^+/+^ culture supernatants (p<0.0001) (Figure 7B). Treatment of LPS/IFN-γ activated macrophages with L-NAME, but not nor-NOHA, largely ablated NO production by both MKP-2^−/−^ and MKP-2^+/+^ macrophages (p<0.0001) (Figure 7B). *T. gondii* tachyzoite infection was found to enhance macrophage arginase-1 expression (Figure 8A) although this was consistently higher in MKP-2^−/−^ than in MKP-2^+/+^ macrophages up to 24 hours post-infection. Increased expression of arginase-1 following macrophage infection with *T. gondii* (Figure 8A) was also reflected in up-regulated enzyme activity at 24 hours post-infection in both MKP-2^−/−^ (p<0.05) and MKP-2^+/+^ macrophages (Figure 8B). Arginase-1 activity was significantly greater in MKP-2^−/−^ than in MKP-2^+/+^ infected macrophages (p<0.001 for non-activated macrophages and p<0.0001 for LPS/IFN–γ activated macrophages) but was effectively and equally inhibited by nor-NOHA in both MKP-2^−/−^ and MKP-2^+/+^ macrophages. Interestingly while L-NAME treatment significantly increased arginase-1 activity in non-activated MKP-2^−/−^ and MKP-2^+/+^ macrophages (p<0.02 for both) and LPS/IFN-γ activated MKP-2^−/−^ cultures (p<0.01) this was not noted in activated MKP-2^+/+^ macrophages (Figure 8B).

## Discussion

The present study demonstrates an important role for MKP-2 in controlling infection with *T. gondii* as infected MKP-2^−/−^ C57BL/6 mice were found to be less able to control parasite growth during both acute and chronic infection as well as displaying increased mortality compared with their wild-type counterparts. The enhanced susceptibility of MKP-2^−/−^ mice was associated with increased tissue arginase-1 expression, generally associated with Th2 responses, and at the same time lowered serum nitrite levels, generally associated with type-1 responses. Nevertheless, increased susceptibility was not associated with any significant modifications of the adaptive immune response between MKP-2 deficient and wild-type mice and the type-1 responses generated in infected MKP-2^−/−^ mice were at least as strong as in their MKP-2^+/+^ counterparts. Rather, the recently identified unique feature of MKP-2 as a negative regulator of macrophage arginase-1 expression and a positive regulator of macrophage iNOS expression [35] would appear to underlie the important role of this member of the dual specific phosphatase family in controlling infection with *T. gondii*. While highlighting the importance of iNOS and NO production in controlling *T. gondii* infection, the present study also uncovered a protective role for arginase-1 in controlling parasite multiplication that can compensate for NO deficiency during early infection.

Generally protective immunity against *T. gondii* is associated with a type-1 response where IFN-γ synergizes with TNF-α to activate macrophages and induce the expression of inducible nitric oxide synthase (iNOS) that catalyzes the formation of nitric oxide (NO) from L-Arginine. While NO can directly kill the parasites [6], [10] some studies have also shown that NO promotes tachyzoite conversion to the much slower dividing bradyzoite form of the parasite through inhibition of mitochondrial and nuclear enzymes essential for parasite respiration [41]. Although iNOS seems to be the predominant pathway used by classically activated macrophages to control *T. gondii* proliferation in tissue culture, the role of NO during *in vivo* infection is less clear. Studies using iNOS-deficient mice have shown that mice lacking iNOS are able to survive and control tachyzoite growth during the acute stage of infection via an IFN-γ dependent mechanism and only succumb during chronic toxoplasmosis [42]. Death was associated with uncontrolled proliferation of tachyzoites in the brain, suggesting that the protective anti-toxoplasmic effect in the brain was iNOS-dependent [42], [43]. Nevertheless, the observation that iNOS deficient mice were able to survive acute infection in an IFN-γ mediated manner suggested that there were alternative pathways other than NO production mediating anti-toxoplasma resistance *in vivo* [44]. In addition, a further IFN-γ and NO independent control of *T. gondii* has been identified in IFN regulatory factor-1 gene deficient mice [45].

In the present study we found that L-NAME treatment of wild-type mice infected with 20,000 type-II Prugniaud-FLUC tachyzoites but not MKP-2^−/−^ mice resulted in enhanced parasite burden and mortality early in infection. This indicated that an NO independent mechanism was playing a protective role in MKP-2^−/−^ mice and controlling parasite growth under conditions where NO generation was being inhibited. Previously induction of the IFN-γ inducible gene IDO has been implicated in mediating some of the IFN-γ dependent NO independent anti-*Toxoplasma* activity [11]. IDO catalyzes the degradation of the essential amino acid L-tryptophan through the kynurenine pathway, thereby depriving the parasite of this essential amino acid [12]. Interestingly the relative contributions of iNOS and IDO to parasite control appear to be tissue specific [12]. More recently immunity-related GTPases (IRGs) have emerged as potent effectors of *T gondii* killing in mice [13], [14]. Thus, murine astrocytes have been shown to have the ability to kill intracellular *T. gondii* independently of iNOS and IDO via IFN-γ inducible IRGs [18]–[20]. Different IRGs have been shown to play roles in controlling acute and chronic infection [16], [17] although the mechanisms through which p47 GTPases confer resistance to *T. gondii* infection have not been determined [21]. However, as no difference in IFN-γ production was noted between infected MKP-2^+/+^ and MKP-2^−/−^ mice infected with *T. gondii* this would suggest GTPase production was not associated with the NO independent resistance demonstrated by MKP-2^−/−^ mice. We have now examined qRT-PCR the expression of the IFN-γ inducible genes LRG47 and IGTP GTPase in infected MKP-2^−/−^ and MKP-2^+/+^ mice and indeed found them comparable. Consequently, this would suggest that an IFN-γ independent mechanism was operating to protect MKP-2^−/−^ mice in the absence of NO production.

Butcher and colleagues (2011) have recently demonstrated that the type-1 strain *T. gondii* parasites deficient in ROP16 have enhanced growth in macrophages, and *in vivo* infection results in increased parasite multiplication and dissemination in the host. This has been associated with the inability of ROP16 deficient parasites to induce STAT-6 mediated arginase-1 expression resulting in maintenance of host cell L-arginine which is exploited by the parasite. It has previously been demonstrated that *T. gondii* is an L-arginine auxotroph [40] and parasite multiplication in the host cell is L-arginine dependent. Previously studies have demonstrated that polymorphisms in ROP-16 from type-2 parasites result in their inability or greatly reduced ability to activate STAT3/6 [46], [47]. However, in the course of the present study these type-2 parasites were shown to increase arginase-1 expression and activity in both MKP-2^+/+^ and MKP-2^−/−^ macrophages. While often thought of as a Th2 associated product of alternative macrophage activation, innate macrophage activation via TLR-4 ligation [48], [49] has also been shown to induce arginase-1 expression. As *T gondii* has been demonstrated to have a number of TLR-4 ligands such as GPI anchors [50] and HSP70 [51], [52] this is the likely source of the STAT-6 independent induction of arginase-1 by type-2 strain parasites as demonstrated in the present study. *In vivo* treatment with nor-NOHA to inhibit arginase-1 activity during the course of the present study resulted in enhanced parasite multiplication in MKP-2^−/−^ mice. This indicated that the enhanced expression of arginase-1 expression in these mice could in some part compensate for iNOS deficiency compared with MKP-2^+/+^ animals.

Many studies suggest that macrophage killing of parasites via iNOS catalyzed NO production is the main mechanism by which *T. gondii* parasite multiplication is controlled [33], [53] and that L-arginine depletion by arginase-1 counter-regulates the effectiveness of iNOS and facilitates parasite growth. Our *in vitro* studies utilizing classically activated BMDM clearly demonstrate that iNOS catalyzed NO production plays the major role in controlling parasite growth and this could be reversed by treatment of cultures with L-NAME. This was irrespective of whether MKP-2^−/−^ or MKP-2^+/+^ macrophages were examined. Interestingly despite producing less NO than activated MKP-2^+/+^ macrophages, activated MKP-2^−/−^ macrophages were equally able to control parasite growth. Indeed inhibition of arginase-1 activity by nor-NOHA treatment did not facilitate parasite growth inhibition in activated MKP-2^−/−^ or MKP-2^+/+^ macrophages, but in fact reversed inhibition of parasite growth early under conditions of activation. This clearly indicates a role for arginase-1 in protection against *T. gondii*. Surprisingly no differences in intracellular parasite growth in MKP-2^−/−^ versus MKP-2^+/+^ macrophages *in vitro* were noted, suggesting the differential weighting of the alternative control mechanisms were compensating for each other over the course of the experiment. Nevertheless, the *in vivo* consequences of MKP-2 deficiency are significant in terms of parasite growth and long-term survival, and this would be in keeping with NO playing a role post-acute infection. It is also likely that cells other than macrophages are contributing to the dysregulated iNOS/arginase-1 expression bias *in vivo* in infected MKP-2^−/−^ mice. Likely candidates would be neutrophils: these have previously been shown to play a significant role in early *T. gondii* infections [54] and have been shown to be major sources of arginase-1 activity *in vivo* and influence the disease process in humans [55]. This is currently under investigation in the murine model.

Our studies on the consequences of MKP-2 deficiency have revealed some fascinating insights into the control of *T. gondii* infection. Firstly NO induction is ultimately of paramount significance in controlling parasite multiplication and host survival. However, in the absence of NO production enhanced arginase-1 is able to in part compensate for this deficiency presumably by starving the parasite of L-arginine as previously demonstrated [31]. Previously it has been suggested that arginase-1 stimulates parasite growth by converting L-arginine to the polyamines needed by the parasite [32] or inhibiting iNOS activity by L-arginine depletion [33]. We could find no evidence for this. Rather we would propose that arginase-1 and iNOS work together to control parasite multiplication by a combination of L-arginine starvation (arginase-1 and iNOS) and NO killing (iNOS).

Overall our results demonstrate that MKP-2 through its ability to reciprocally modulate arginase-1 and iNOS expression is a key regulator in L-arginine metabolism and consequently this has clear consequences for the control of intracellular parasites. Furthermore as arginase-1 has also been shown to have potent T cell modulatory effects [55], [56] MKP-2 influences are likely to have significant consequences for inflammatory disease and cancers where arginase-1 and iNOS have already been identified as key players [57]–[59]. These observations identify manipulation of MKP-2 expression or activity as a significant target for future therapeutic strategies.

## Supporting Information

Figure S1 ***In vitro*** **assessment of bioluminescence of** ***T. gondii*** **prior to murine infection and assessment of optimal** ***in vivo*** **imaging time.** Prior to infections being carried out *T. gondii* tachyzoites were assessed for their ability to luminesce (A). This was carried out as a quality control step prior to every infection. The optimal time to image mice following luciferin injection (B) was determined. MKP-2^+/+^ mice infected with 20,000 FLUC tachyzoites were imaged for 1 minute, every 5 minutes post luciferin injection. A consistent maximum signal was evident from 15–35 minutes post luciferin injection. 20 minutes was therefore chosen for all future experiments.
(TIF)

Figure S2 **MKP-2^−/−^ mice have an increased parasite burden and display no differences in immune regulated GTPases associated with control of** ***T. gondii*** **infection.** On day 7 post infection, peritoneal exudates from MKP-2^+/+^ and MKP-2^−/−^ mice infected with FLUC *T. gondii* were assessed for parasite burden by quantitative real-time PCR (qRT-PCR) for parasitic SAG1 (A) There were no parasites detected by qRT-PCR in the tissues at this stage but by day 10 post-infection increased parasite burdens were noted in the tissues including the lungs of MKP-2^−/−^ mice (B). Splenocytes from infected MKP-2^+/+^ and MKP-2^−/−^ were analysed by qRT-PCR for expression of murine LRG47 and IRGP (C). Each value represents 4 replicates ±SEM. * P<0.05. Results are from 2 independent experiments
(TIF)

Figure S3 **Total numbers of cytokine producing T cells.** Splenocytes from *T. gondii* infected mice were re-stimulated and analysed using flow cytometry. Cells were surface-stained for CD3, CD4 and CD8 and intracellular for IFN-γ and TNF-α. Populations of CD3^+^ CD4^+^ T cells (A) and CD3^+^ CD8^+^ T cells (B) single or double positive for IFN-γ and TNF-α were determined and normalized to their respective isotype control. Total numbers of cytokine producing cells were calculated by multiplying the percentage value with the total cell number for the individual spleen. Each bar represents the mean of four animals per experimental group ± SEM. All experiments were carried out on at least two occasions.
(TIF)

Figure S4 **Infected MKP-2^+/+^ and MKP-2^−/−^ mice have similar numbers of IFN-γ producing NKT cells.** Intraperitoneal exudates from *T. gondii* infected mice were surface stained for CD3 and Pan-NK and intracellular for IFN-γ and analysed by flow cytometry. Viable, Pan-NK positive cells were analysed for the presence of CD3 and distinguished between CD3^+^, Pan-NK^+^ (NKT cells) and CD3^−^, Pan-NK^+^ (NK cells) populations and values for IFN-γ positive cells were normalized to their respective isotype control. Since NK cells did not produce any IFN-γ only NKT cells are presented. Shown are the %IFN-γ^+^ cells from all PanNK^+^ cells (A) and %IFN-γ^+^ NKT cells from the total cell population (B). Each bar represents the mean of four animals per experimental group ± SEM.
(TIF)

Table S1 **Primer sequences.** Oligonucleotide sequences of primers used for analysis of gene expression by Real-Time PCR.
(TIF)
