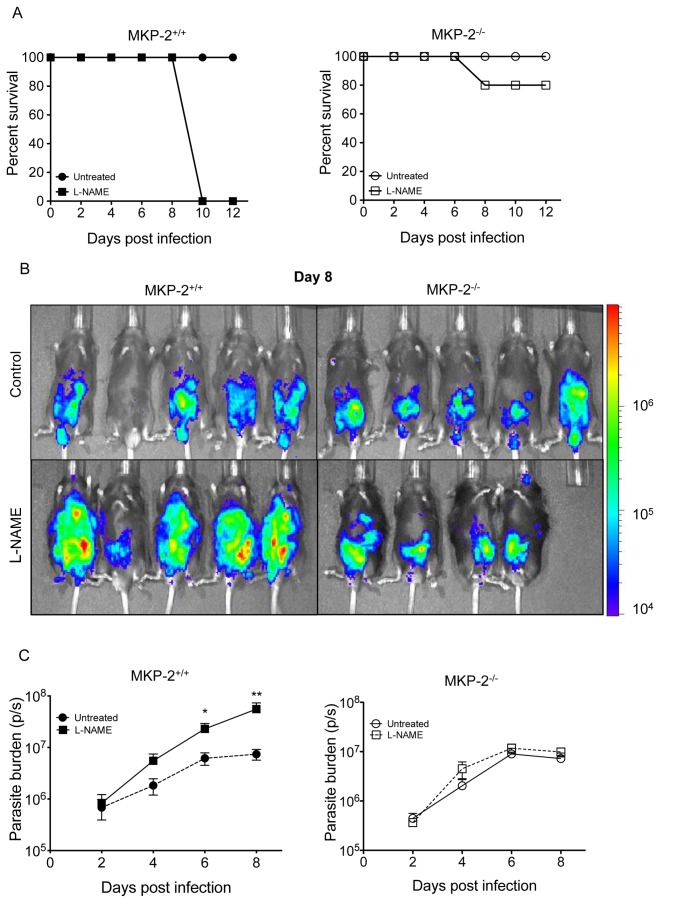# Correction: MAP Kinase Phosphatase-2 Plays a Key Role in the Control of Infection with *Toxoplasma gondii* by Modulating iNOS and Arginase-1 Activities in Mice

**DOI:** 10.1371/annotation/408bc3ff-0000-4e72-b4fb-839c6c848178

**Published:** 2013-09-23

**Authors:** Stuart Woods, Juliane Schroeder, Helen A. McGachy, Robin Plevin, Craig W. Roberts, James Alexander

There is an error in the control group panel in part B of Figure 5. This panel is a duplicate of the control group panel in part B of Figure 6. Please download the correct version of Figure 5 at the following link: 

**Figure:**