# Supplementary figures and images for: MAP Kinase Phosphatase-2 Plays a Key Role in the Control of Infection with Toxoplasma gondii by Modulating iNOS and Arginase-1 Activities in Mice
Source: PLoS Pathog. 2013 Aug 15;9(8):e1003535. doi: 10.1371/journal.ppat.1003535 (PMC3744406; doi:10.1371/journal.ppat.1003535)

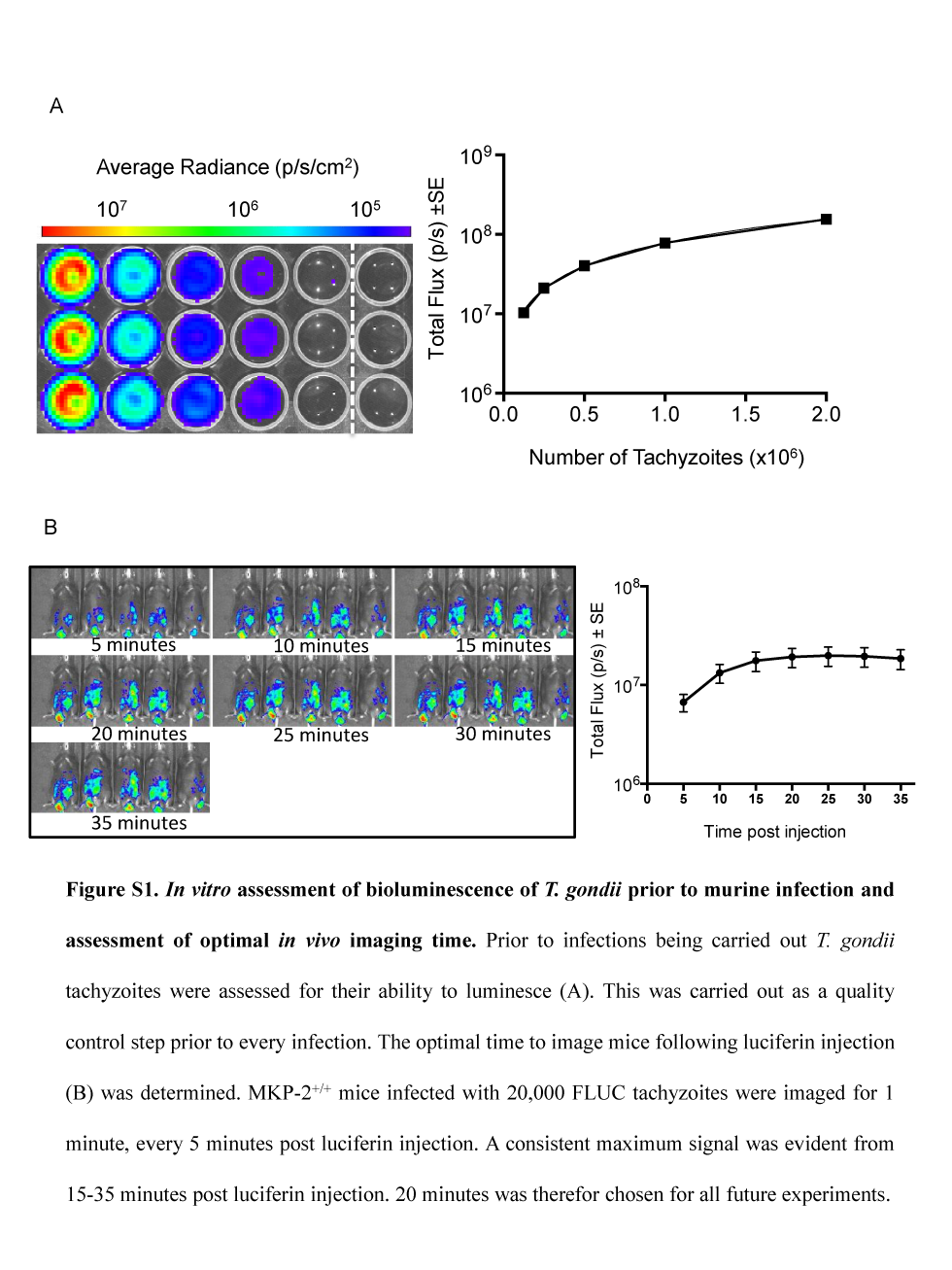

Supplement: Figure S1 — In vitro assessment of bioluminescence of T. gondii prior to murine infection and assessment of optimal in vivo imaging time. Prior to infections being carried out T. gondii tachyzoites were assessed for their ability to luminesce (A). This was carried out as a quality control step prior to every infection. The optimal time to image mice following luciferin injection (B) was determined. MKP-2+/+ mice infected with 20,000 FLUC tachyzoites were imaged for 1 minute, every 5 minutes post luciferin injection. A consistent maximum signal was evident from 15–35 minutes post luciferin injection. 20 minutes was therefore chosen for all future experiments. (TIF) [file ppat.1003535.s001.tif]

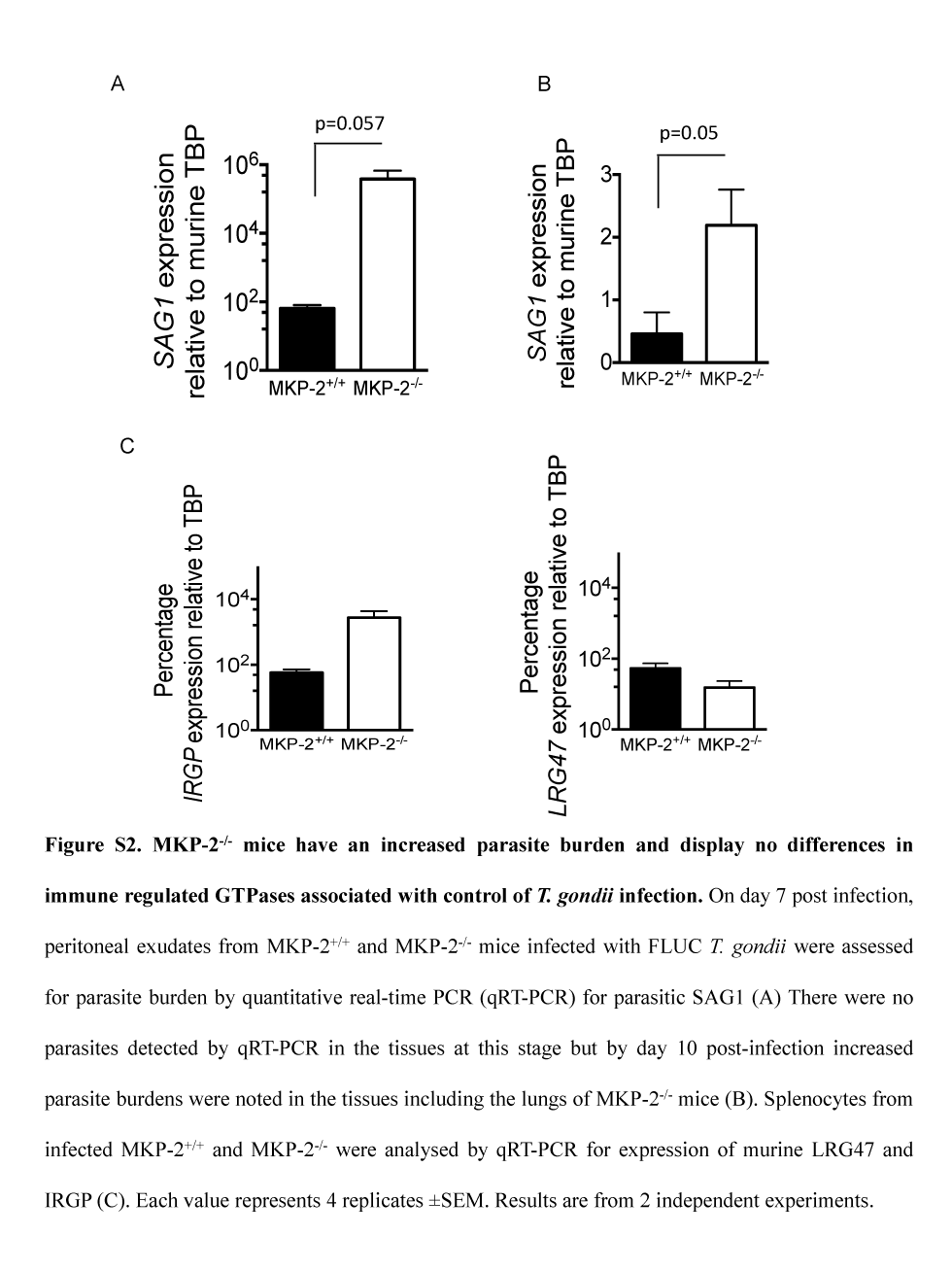

Supplement: Figure S2 — MKP-2−/− mice have an increased parasite burden and display no differences in immune regulated GTPases associated with control of T. gondii infection. On day 7 post infection, peritoneal exudates from MKP-2+/+ and MKP-2−/− mice infected with FLUC T. gondii were assessed for parasite burden by quantitative real-time PCR (qRT-PCR) for parasitic SAG1 (A) There were no parasites detected by qRT-PCR in the tissues at this stage but by day 10 post-infection increased parasite burdens were noted in the tissues including the lungs of MKP-2−/− mice (B). Splenocytes from infected MKP-2+/+ and MKP-2−/− were analysed by qRT-PCR for expression of murine LRG47 and IRGP (C). Each value represents 4 replicates ±SEM. * P<0.05. Results are from 2 independent experiments (TIF) [file ppat.1003535.s002.tif]

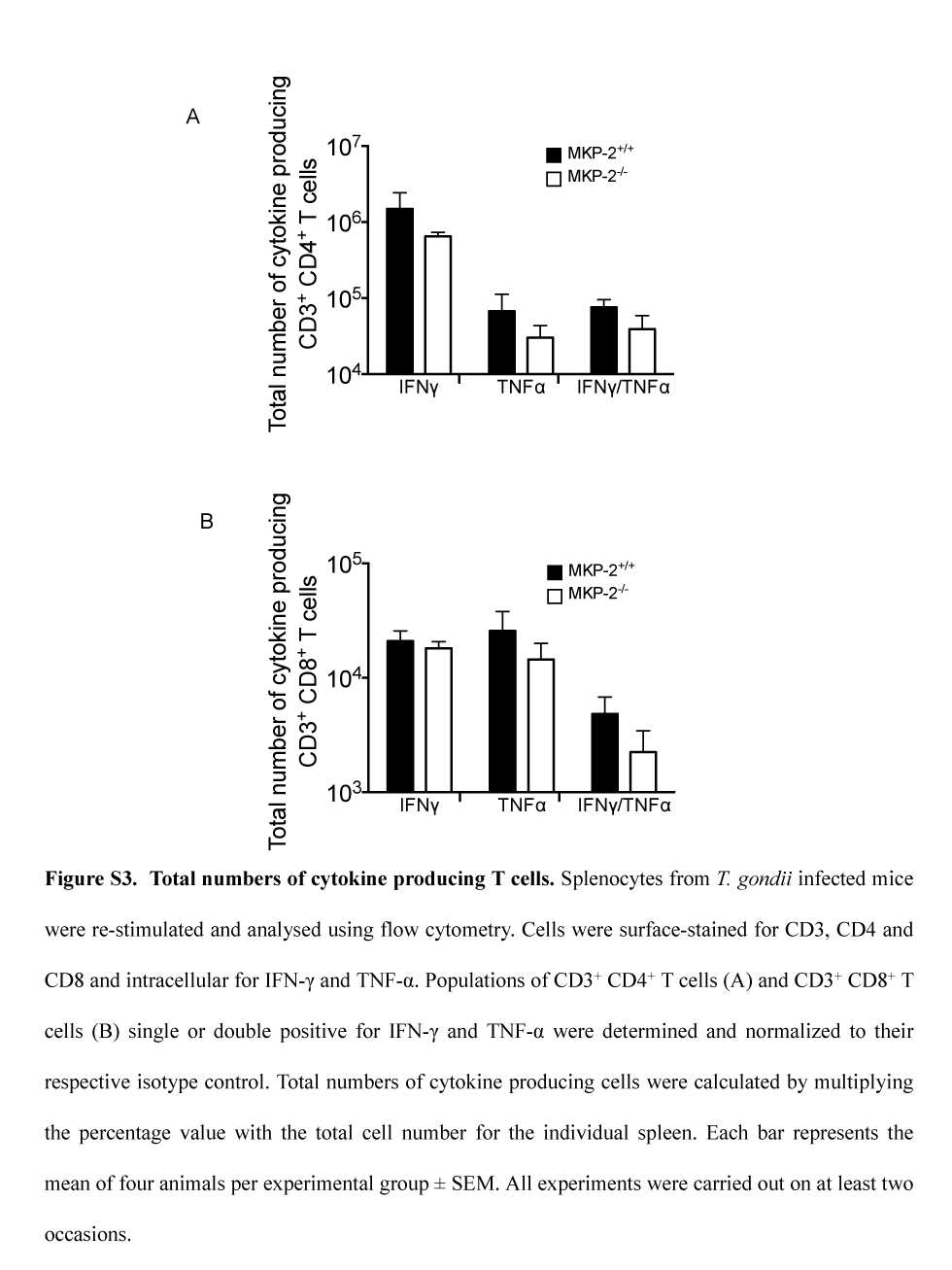

Supplement: Figure S3 — Total numbers of cytokine producing T cells. Splenocytes from T. gondii infected mice were re-stimulated and analysed using flow cytometry. Cells were surface-stained for CD3, CD4 and CD8 and intracellular for IFN-γ and TNF-α. Populations of CD3+ CD4+ T cells (A) and CD3+ CD8+ T cells (B) single or double positive for IFN-γ and TNF-α were determined and normalized to their respective isotype control. Total numbers of cytokine producing cells were calculated by multiplying the percentage value with the total cell number for the individual spleen. Each bar represents the mean of four animals per experimental group ± SEM. All experiments were carried out on at least two occasions. (TIF) [file ppat.1003535.s003.tif]

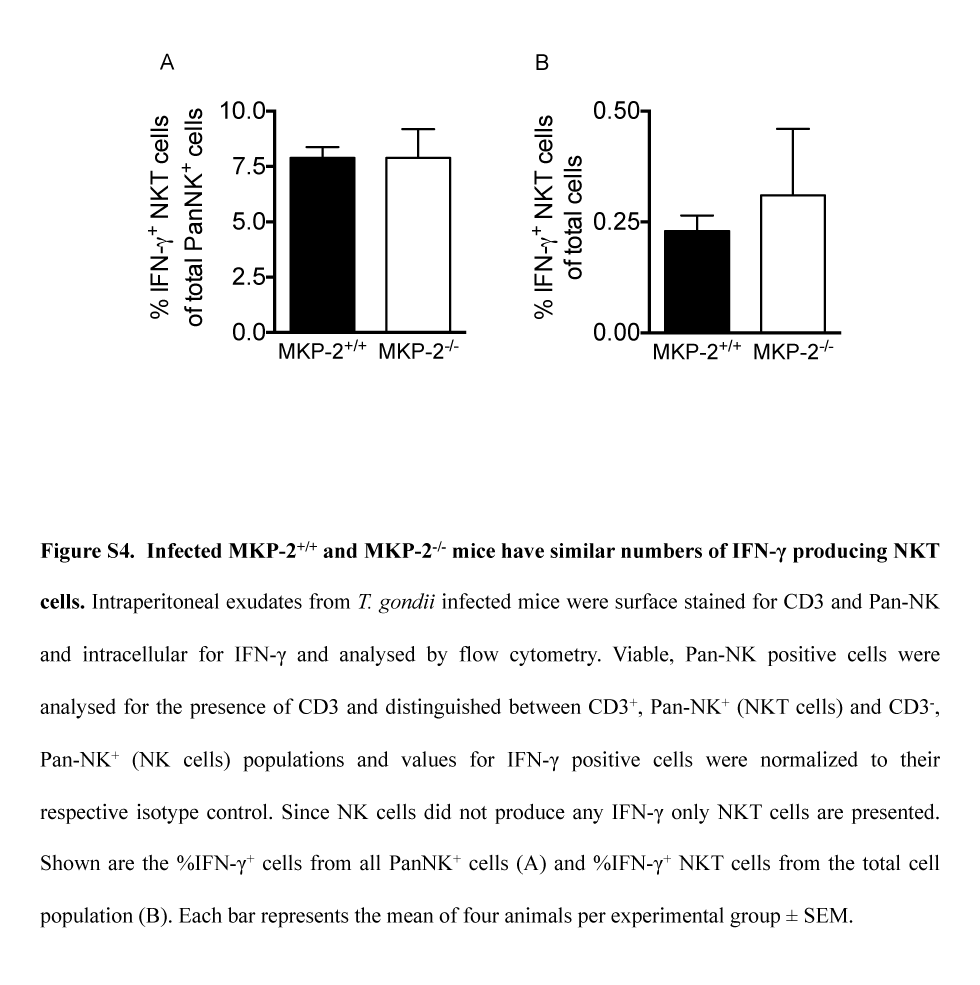

Supplement: Figure S4 — Infected MKP-2+/+ and MKP-2−/− mice have similar numbers of IFN-γ producing NKT cells. Intraperitoneal exudates from T. gondii infected mice were surface stained for CD3 and Pan-NK and intracellular for IFN-γ and analysed by flow cytometry. Viable, Pan-NK positive cells were analysed for the presence of CD3 and distinguished between CD3+, Pan-NK+ (NKT cells) and CD3−, Pan-NK+ (NK cells) populations and values for IFN-γ positive cells were normalized to their respective isotype control. Since NK cells did not produce any IFN-γ only NKT cells are presented. Shown are the %IFN-γ+ cells from all PanNK+ cells (A) and %IFN-γ+ NKT cells from the total cell population (B). Each bar represents the mean of four animals per experimental group ± SEM. (TIF) [file ppat.1003535.s004.tif]

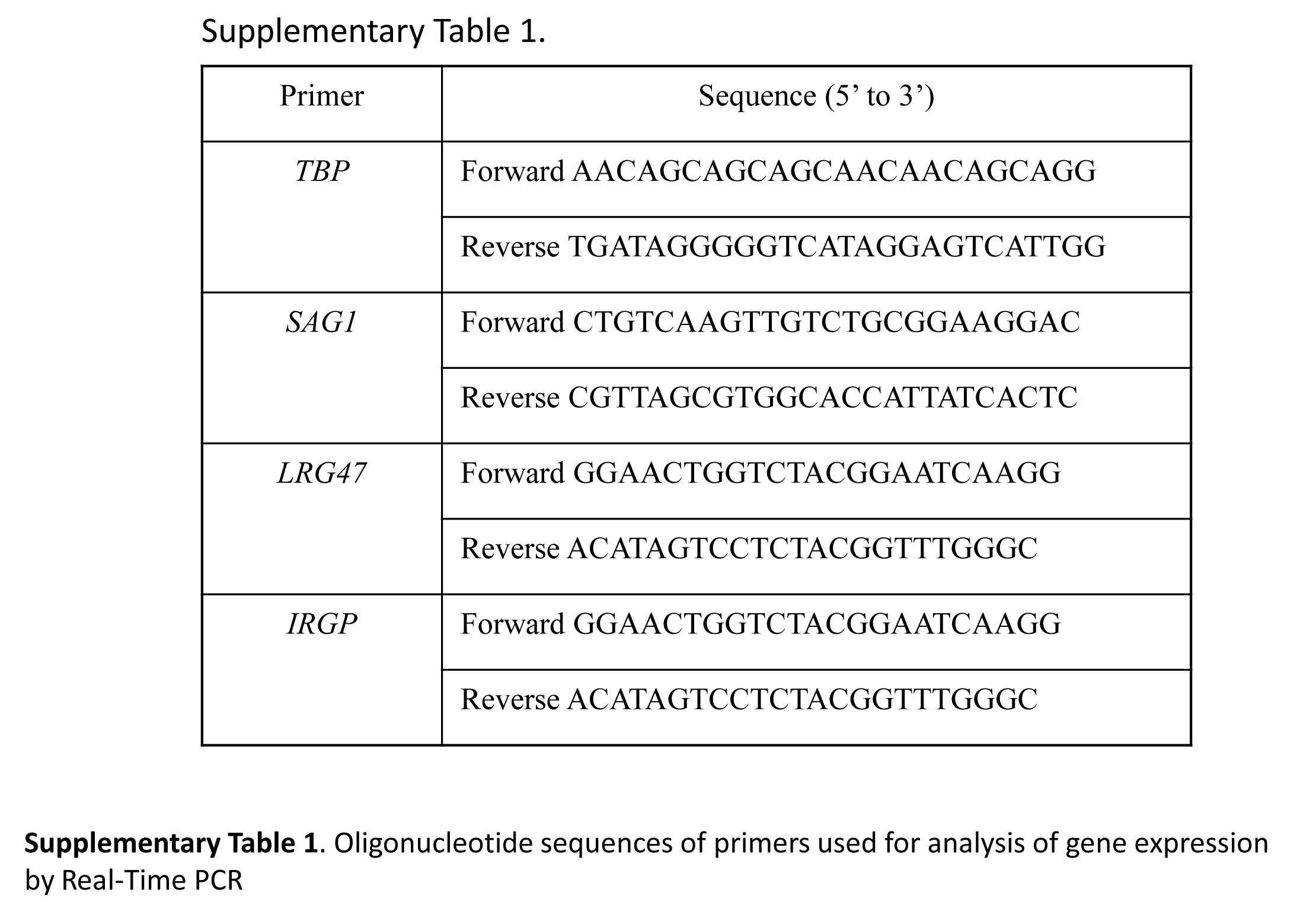

Supplement: Table S1 — Primer sequences. Oligonucleotide sequences of primers used for analysis of gene expression by Real-Time PCR. (TIF) [file ppat.1003535.s005.tif]
